# Books and Magazines

**Published:** 1907-01

**Authors:** 


### Books and Magazines.

The Medical Epitome Series: Kiepe's Materia Medica and Therapeutics.—A Manual for Students and Physicians attending post-graduate courses. By Edward J. Kiepe, Professor of Materia Medica in the Department of Pharmacy, and Adjunct Professor of Materia Medica and Pharmacology in the Medical Department, University of Buffalo. In one 12mo volume of 265 pages. Cloth, $1, net. Lea Brothers & Co., Publishers, Philadelphia and New York, 1906.

The Medical Epitome Series when complete will consist of twenty-three volumes. This is the twentieth, leaving but three more to complete the set. It is easy for students, and practitioners as well, to post themselves to date for examinations or practical purposes, or attending post-graduate courses, by reading these authoritative little books. They are written by professors or teachers in colleges of high standing, and the subjects are treated in a manner as clear, thorough and interesting as the necessary limits of space will permit.

Poker Jim, Gentleman—A Story of California, and Other Sketches. By G. Frank Lydston, M. D. Monarch Publishing Company, Chicago. Illustrated. Price, $1.

These stories are written in Dr. Lydston's characteristic, fluent, breezy and very fascinating style, and will, therefore, interest the reader from start to finish. The "other sketches" are much the best; some of them reminding one, first, of Poe, and then of Balzac.

"Poker Jim, Gentleman," is the well-known, handsome, dare-devil, toney gambler of the mining camps, shooting on short notice, handy and unerring with his "gun," and getting into scraps, always. He is of the John Oakhurst type, made immortal by Bret Harte. These sketches seem to be reminiscent of Dr. Ldyston's experiences. Buy the book and read it.

---

Practical Medicine Series. The Year Book Co., Publishers, 40 Dearborn St., Chicago, 1906. The series consists of ten volumes, each year, issued at about monthly intervals, and covering the entire field of medicine and surgery. Each volume being complete for the year prior to its publication on the subject of which it treats. Price per volume, $1.25. Price of the series of ten volumes, $10.

We have received volumes 4 to 9, inclusive.

Volume 4—Gynecology. Dudley, Bachelle.

Volume 5—Obstetrics. Lee.

Volume 6—General Medicine. Billings, Salisbury.

Volume 7—Pediatrics and O. P. Surgery. Ridlow.

Volume 8—Therapeutics, Practice of Medicine, Climatology, Forensic Medicine.

Volume 9—Physiology, Pathology, and Bacteriology. Evans and Gehrman.

These are all gleaned from the late medical journals and are more up-to-date than it is possible for any text-book to be.

SAUNDERS' POCKET MEDICAL FORMULARY. By William M. Powell, M. D., author of "Essentials of Diseases of Children"; Member of Philadelphia Pathologic Society. Containing 1831 formulas from the best known authorities. With an appendix containing Posologic Tables, Formulas and Doses for Hypodermic Medication, Poisons and their Antidotes, Diameters of the Female Pelvis and Fetal Head, Obstetric Table, Diet-lists, Materials and Drugs used in Antiseptic Surgery, Treatment of Asphyxia from Drowning, Surgical Remembrancer, Tables of Incompatibles, Eruptive Fevers, etc., etc. Eighth edition, adapted to the new (1905) Pharmacopeia. Philadelphia and London: W. B. Saunders Company, 1906. In flexible morocco, with side index, wallet and flap, $1.75, net.

---

RETINOSCOPY (Shadow Test) in the Determination of Refraction at One Meter Distance, With the Plane Mirror. By James Thorington, A. M., M. D., Professor Diseases of Eye, Philadelphia Polyclinic, etc., etc. Fifth edition, revised and enlarged. Fifty-four illustrations, ten colored. P. Blakiston's Son & Co., Philadelphia, 1906. Price, $1.

Previous editions of this valuable little hand-book have been received with much praise and commendation at the hands of the medical press. Our readers will welcome this improved edition. It is indispensable to oculists.

---

SECOND REPORT OF THE WELCOME RESEARCH LABORATORIES AT GORDON MEMORIAL COLLEGE, KHARTOUM. Andrew Balfour, M. D., B. Sc., F. R. C. P., Edinburg; D. P. H. Camb, Director. Health Office at Khartoum, etc. From the Department of Education, Sudan Government, Khartoum, 1906.

Talk about Darkest Africa, when the English government has established such an institution as this on the spot where, a few short years ago the great Gordon Pasha, "Chinese Gordon," with the entire English army was massacred by the naked savages of the Mahdi. Mr. Welcome, the founder of this great Research Laboratory, is an American. He is of the great English firm of Burrows, Welcome & Co., London. The object is the study of disease-producing insects, worms and microbes, the chief factors in tropical disease. The book is beautifully illustrated with colored pictures of the flies and bugs and things that cause so much trouble, and the study throws much light on the pathology and treatment of the diseases common to the tropics. It will strike American readers with surprise to know that in Khartoum away over in the Sudan—in Darkest Africa, on the Blue Nile—the mosquito has been practically exterminated. Uncle Sam should send some students over there to study pathogenic insects and things. Thanks, very much, for this valuable book.

Medical Guides and Monograph Series—Golden Rules of Pediatrics, Aphorisms, Observations and Precepts on the Science and Art of Pediatrics; Giving Practical Rules for Diagnosis and Prognosis, the Essentials of Infant Feeding, and the Principles of Scientific Treatment. By John Zahorsky, A. B., M. D., Clinical Professor of Pediatrics, Washington University Medical Department, St. Louis, Mo., etc., etc., etc. Editor *St. Louis Courier of Medicine,* etc. Author of "Baby Incubators," etc. With an introduction by E. W. Saunders, M. D., Professor of Diseases of Children and Clinical Midwifery, Washington University, St. Louis, 1906. The C. V. Mosby Medical Book Co. Price, $3.

The title is descriptive of the character and contents of the book.

---

The Medical Epitome Series—Pathology, General and Special; A Manual for Students and Practitioners. By Jno. Stenhouse, M. A., B. Sc., Edinburg; M. B., Toronto; Formerly Demonstrator of Pathology, University of Toronto, Toronto Can.; and Jno. Ferguson, M. A., M. D., Toronto; Senior Physician Western Hospital, etc., etc. Series edited by Victor Cox Pedersen, A. M., M. D., Lecturer in Surgery at New York Polyclinic Medical School and Hospital, etc., etc. Illustrated with sixteen engravings and a colored plate. Price, $1. Lea Brothers & Co., Philadelphia and New York, 1906.

This manual will be found very useful for ready reference. It is more than a resume of text-books, as more recent matter appearing in medical journals has been incorporated.

Diseases of the Digestive System. Edited by Frank Billings, M. D., Professor of Medicine, University of Chicago; Professor of Medicine and Dean of Faculty, Rush Medical College, Chicago, Ill. D. Appleton & Co., New York. Price not given.

This is an authorized translation from *Die Deutsche Klinik* and the third volume in Modern Clinical Medicine, the first volume, "Infectious Diseases," edited by J. C. Wilson, A. M., M. D., Philadelphia; the second volume, "Diseases of Metabolism and of the Blood, Animal Parasites, Toxicology," edited by Richard C. Cabot, M. D.

This third volume should receive a very warm reception, for today *diseases of the digestive tract* stand in the forefront of subjects which interest the practitioner and the surgeon. Much that was formerly theoretical in relation to this subject has now become almost absolute knowledge.

This volume includes articles from many of the most eminent men of Europe, specialists in internal medicine and in diseases of the digestive tract, such as Rosenheim, Fleiner, Leo, Strauss, Reigel, Ewlad, Boas, Hirschfeld, Osler, Minkowski, Stadelmann, Kraus, Neusser, Vierordt, Strausburger, Hoppe-Seyler, and Nothnagel.

Subjects are treated very fully and at the same time in a concise and practical manner. Modern methods of examination, including physical and chemical measures, are clearly set forth. The diagnosis of the various diseases is fully discussed, and the treatment, including the dietary, is satisfactorily full and complete.

This work will be found a valuable complement to the two preceding volumes. Such names as Wilson, Cabot, and Billings as editors give assurance of the worth of this series, which should be in the library of every up-to-date physician.

---

Transactions of the Fourth Annual Conference of State and Territorial Health Officers With the U. S. Public Health and Marine Hospital Service, Washington, D. C., May 23, 196.

We have received two copies of this work issued by the U. S. Public Health and Marine Hospital Service, Government Printing Office, Washington, D. C. The topics discussed were "Water Pollution," "Railway Car Sanitation," and "Prevention of Tuberculosis." This volume is a valuable symposium on those important subjects and should be preserved for reference by all who are interested in the public health of the nation. Most of the American States and Territories were represented at the conference, *except Texas.*

THE PHYSICIAN'S VISITING LIST FOR 1907. P. Blakiston's Son & Co., Philadelphia. Fifty-sixth year of its publication. With special memoranda; 25 patients per week. Flexible black morocco, a convenient pocket size and very neat in appearance. Price, $1.

This little favorite is well known to the medical profession. It is only necessary to announce that it is ready; and to say that the dose-table has been revised in accordance with the new U. S. Pharmacopeia (1900).

---

PRACTICAL DERMATOLOGY—A Condensed Manual of Diseases of the Skin; Designed for the Use of Students and Practitioners of Medicine. By Bernard Wolff, M. D., Clinical Professor of Diseases of the Skin in Atlanta College of Physicians and Surgeons. Editor *Atlanta Journal-Record of Medicine;* Ex-President of the Fulton County (Ga.) Society of Medicine; Ex-Secretary of the Georgia State Commission on Tuberculosis, etc. Illustrated. Cleveland Press, Chicago, 1906.

Our confrere of the Atlanta *Journal-Record of Medicine* has issued a very creditable and useful *epitome* of Diseases of the Skin, and is to be congratulated. In this volume he presents "in miniature the salient features of diseases of the skin." It will be found most useful for hurried reference and save wading through the larger and standard works. Price not given.

